# Voltammmetric Assessment and Examination of the Interactions between Levetiracetam and DNA: Experimental Research, Molecular Docking, and Modeling Studies

**DOI:** 10.1002/open.202500191

**Published:** 2025-07-03

**Authors:** Abdullah Al Faysal, Pelin Şenel, Taner Erdoğan, Ayşegül Gölcü

**Affiliations:** ^1^ Department of Chemistry Istanbul Technical University Faculty of Sciences and Letters Maslak 34469 Istanbul Türkiye; ^2^ Department of Chemistry and Chemical Processing Technologies Kocaeli University Kocaeli Vocational School 41140 Kocaeli Türkiye

**Keywords:** drug quantifications, groove binding, levetiracetam, molecular modeling, voltammetry

## Abstract

Levetiracetam (LEV) is an innovative antiepileptic medication utilized for the management of diverse seizure types associated with epilepsy. The present study aims to elucidate the molecular interaction mechanisms between LEV and fish sperm DNA (dsDNA) through a combination of spectroscopic techniques, viscosity measurements, and molecular docking analyses. Spectroscopic investigations, including UV absorption and fluorescence, confirm the formation of a complex between LEV and dsDNA. The groove binding process is indicated by the measured binding constant. Viscosity, dye‐displacement test, and DNA thermal denaturing investigations are used to confirm these results. Docking studies further verify the results, which show that LEV is linked to the minor groove of dsDNA. Furthermore, an LEV–dsDNA biosensor for low‐concentration LEV detection using the differential pulse voltammetry technique is created. A sensitive determination of LEV in pH 4.80 acetate buffer is made possible by the voltammetric examination of the peak current drop in the deoxyguanosine (dGuo) oxidation signals that resulted from the interaction between LEV and dsDNA. The oxidation signals of dGuo demonstrate a linear correlation within the concentration range of 2.5–20 μM LEV. The limit of detection and limit of determination are found to be 0.70 and 2.31 μM, respectively.

## Graphical abstract

This research introduces, for the first time, a novel, efficient, cost‐effective, and highly sensitive approach for the quantification of levetiracetam (LEV) through a differential pulse voltammetry technique based on its interaction with DNA. The method capitalizes on the changes observed in the oxidation current peak of the deoxyguanosine base, which are influenced by varying concentrations of LEV.

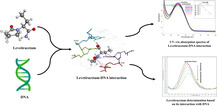

## 1 Introduction

Epilepsy is a long‐lasting neurological condition marked by the occurrence of repeated, untriggered seizures.^[^ ^1^ ^]^ Epilepsy impacts around 50 million individuals globally.^[^ ^2^ ^]^ Epilepsy has the potential to lead to neurological impairment, significant physical harm, and in certain cases, fatal outcomes.^[^ ^3^ ^]^ In acute care settings, seizures must be managed quickly. The primary objective in managing a patient experiencing a seizure is to halt all seizure activity as swiftly as possible. This prompt intervention is crucial in minimizing the associated disability and death that can arise from the initial event, as well as mitigating physiological stress and potential damage to the central nervous system.^[^ ^4^ ^]^ Levetiracetam (LEV) has gained widespread acceptance in clinical practice since its launch in 1999, establishing itself as the primary therapeutic option for a variety of epilepsy syndromes and clinical situations.^[^ ^5^ ^]^ By attaching to the synaptic vesicle glycoprotein 2A, LEV modulates the discharge of neurotransmitters into the body.^[^ ^6^, ^7^ ^]^ LEV does not undergo metabolism via the liver's cytochrome P450 isoenzymes, which significantly reduces the likelihood of drug interactions commonly associated with other antiepileptic medications. Furthermore, the adverse effects associated with LEV are relatively minimal, with reported symptoms including asthenia, dizziness, and alterations in behavior. Routine monitoring of therapeutic levels is typically unnecessary for LEV, and the potential for toxicity remains low.^[^ ^4^, ^8^, ^9^ ^]^ LEV has received approval for use as an adjunctive treatment for both focal and generalized myoclonic seizures, as well as tonic–clonic seizures. Additionally, it is sanctioned for monotherapy in the management of focal seizures and for the treatment of status epilepticus.^[^ ^10^ ^]^

Deoxyribonucleic acid (DNA) serves as the fundamental genetic substance that governs the various cellular functions and is critical for the transmission of hereditary data essential for the growth and operation of an organism.^[^ ^11^ ^]^ Many substances bind to DNA, which is generally acknowledged as the primary molecular target for anticancer medications^[^ ^12^ ^]^ to exhibit their therapeutic activities. This understanding serves as a foundation for the innovation and identification of new treatments that possess considerable effectiveness. Molecules that engage with DNA often alter the processes of DNA replication and transcription, leading to cellular death and apoptosis.^[^ ^13^, ^14^ ^]^ Therefore, it is essential to comprehend how drug compounds bind to biomacromolecules like DNA in order to develop novel pharmaceuticals for efficient treatments.^[^ ^15^ ^]^ Compounds can interact with DNA through various mechanisms, including covalent binding, noncovalent interactions, or a combination of both. Noncovalent interactions, such as hydrogen bonding, electrostatic forces, and hydrophobic effects, play a crucial role in processes like intercalation and binding within the major and minor grooves of the DNA structure. Groove binders infiltrate the DNA groove, leading to subtle modifications in the DNA conformation, while intercalators engage with the DNA by positioning an aromatic planar chromophore between adjacent base pairs. Typically, the binding of small molecules to the DNA grooves is most prevalent at guanine–cytosine sites within the major groove.^[^ ^16^, ^17^ ^]^

Various analytical techniques, including spectroscopies, chromatography, Fourier‐transform infrared spectroscopy, and electrochemical methods, are employed to elucidate the binding mechanisms between tiny molecules and DNA. Additionally, molecular docking serves as a computational approach to simulate and characterize the specific interactions involved in this binding process.^[^ ^18^, ^19^, ^20^, ^21^, ^22^ ^]^ Generally, the favored methodologies for such analyses are spectroscopic and electrochemical techniques. UV–vis spectroscopy stands out as a highly effective method widely employed to investigate the interactions between DNA and pharmaceutical compounds.^[^ ^23^ ^]^ Fluorescence spectroscopy presents numerous promising applications within the realm of research focused on DNA–drug interactions. This technique is noninvasive and has demonstrated a high level of sensitivity, enabling researchers to effectively characterize the interactions between DNA and various drugs.^[^ ^24^ ^]^ Electrochemical methods present distinct benefits compared to traditional analytical techniques, primarily due to their cost‐effectiveness, rapid response times, specificity, sensitivity, and compatibility with complex biological matrices. Under appropriate conditions, the electroactive properties of adenine and guanine nucleotides within the DNA framework can be harnessed in electrochemical techniques to evaluate the interactions between drugs and DNA.^[^ ^25^ ^]^

This research explored the binding mechanism of LEV to dsDNA through various spectroscopic techniques, including UV–visible spectroscopy, fluorescence analysis, viscosity evaluations, and voltammetric studies. These methodologies offer a thorough understanding of the binding properties of LEV with dsDNA, detailing aspects such as particular binding techniques, binding constants, and binding strengths. A thermodynamic analysis conducted at three distinct temperatures provides valuable insights into the forces driving this interaction. Molecular docking studies were carried out to elucidate the binding method and further confirm the experimental findings. Ultimately, through the application of voltammetry, we developed a thoroughly validated method for quantifying LEV in a tablet formulation (Keppra 250 mg). With a limit of quantification of 2.31 μM, the results obtained with this method are a true replacement for previously published chromatographic and colorimetric techniques.^[^ ^26^, ^27^, ^28^ ^]^

## 2 Results and Discussion

### 2.1 UV Absorption Spectroscopic Study

**Figure 1:**
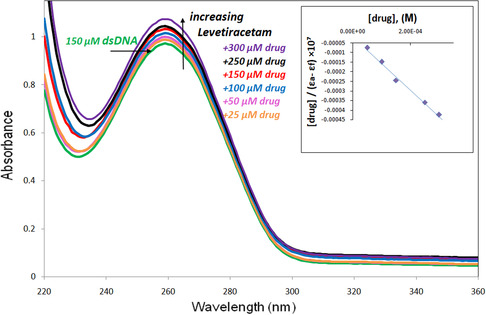
The UV–vis absorption spectra of dsDNA (150 μM) and LEV (from 25 to 300 μM) mixture (in 50 mM Tris–HCl and buffer at pH 7.4).

Absorption spectroscopy is widely employed to investigate the binding interactions between small molecules and DNA, owing to its accessibility and heightened sensitivity. Variations in both the intensity and position of absorption peaks can signify the establishment of secondary complexes within the solution, thereby providing insights into the underlying mechanisms of interaction.^[^ ^29^ ^]^ The interaction between DNA and small molecules is widely recognized to induce alterations in absorbance characteristics and/or the positioning of spectral peaks.^[^ ^30^ ^]^ Small molecules that intercalate into DNA typically induce both hypochromism and bathochromic shift in the absorption peak of the DNA. When small molecules interact with DNA through an electrostatic binding mechanism, a hyperchromic effect is observed, indicating changes in the DNA conformation. Conversely, when small molecules bind to DNA via groove binding, the resulting effects can vary, leading to either hypochromic or hyperchromic responses, along with minimal or no shift in the peak position.^[^ ^31^ ^]^ The UV spectra of dsDNA in the presence of LEV were analyzed at a temperature of 298 K. The absorption spectra depicted in **Figure** **1** illustrate the behavior of 150 μM dsDNA as the concentration of LEV is varied from 25 to 300 μM. Notably, an increase in LEV concentration correlates with a hyperchromic effect observed at 260 nm in the absorption profile of dsDNA.

The Benesi–Hildebrand equation (Equation 4) was applied in this investigation to ascertain the binding affinity (*K*_b_) of LEV with the dsDNA.^[^ ^32^ ^]^

(1) $$\frac{\left[\text{dsDNA}\right]}{\varepsilon_{a}{\text{‐}\varepsilon }_{f}}\text{ = }\frac{\left[\text{dsDNA}\right]}{\varepsilon_{b}{\text{‐}\varepsilon }_{f}}\text{+ }\frac{1}{K_{b}{(\varepsilon }_{b}{\text{‐}\varepsilon }_{f})}$$

In this context, [DNA] signifies the concentration of dsDNA, while *ε*_a_ represents the extinction coefficient linked to the absorption signal at different dsDNA concentrations. Furthermore, *ε*_f_ indicates the extinction coefficient of the unbound LEV, and *ε*_b_ denotes the extinction coefficient of LEV when it is fully associated with dsDNA. The plots show the correlation between [DNA]/(*ε*_a_–*ε*_f_) and [DNA], and the binding constant (*K*_b_) is determined using the slope and intercept ratio, as shown in Figure 1. The computed binding constant is 3.22 × 10^4^ ± 0.14 M^−1^, which is in good agreement with values reported for commonly used groove‐binding compounds. The computed *K*_b_ is particularly distant for conventional intercalators (10^6^–10^7^ M^−1^), but it is within the range of those groove binders, indicating that LEV binds to the DNA helix in a groove.

### 2.2 Analysis of Thermodynamic Parameters

The analysis of enthalpy changes (ΔH) and entropy changes (ΔS) allows for the classification of interactions between these compounds and DNA into three distinct categories. When ΔH is more significant than zero and ΔS is also greater than zero, the interactions are primarily driven by hydrophobic forces. Conversely, when ΔH is less than zero and ΔS is less than zero, the interactions can be attributed to van der Waals forces or hydrogen bonding. Additionally, when ΔH is less than zero while ΔS is more significant than zero, the interactions are indicative of electrostatic forces. A crucial aspect of determining thermodynamic parameters is the relationship between the binding constant and temperature, which can be effectively represented through a linear plot of ln *K*_b_ against 1/T.^[^ ^33^ ^]^ The values of the thermodynamic parameters are presented in **Table** **1**. The observed negative values for enthalpy changes and the positive values for entropy changes indicate that electrostatic forces primarily facilitate the interaction between LEV and dsDNA. Furthermore, the negative ΔG values suggest that the interaction between LEV and dsDNA occurs spontaneously.^[^ ^34^ ^]^

**Table 1:** Thermodynamic parameters of LEV–dsDNA interaction.

T (K)	*K*_b_ (M^−1^)a)	Δ*G* (kJ/mol)	Δ*H* (kJ/mol)	Δ*S* (J/mol K)a)
288.15	3.71 × 10^4^ ± 0.21	–25.18	–6.70	+64.11
298.15	3.22 × 10^4^ ± 0.14	–25.82		
308.15	3.12 × 10^4^ ± 0.13	–26.46		

a) The findings were reported as the average and RSD for each test, which was conducted on three separate occasions.

### 2.3 DNA Melting Temperature (*T*_m_) Study

**Figure 2:**
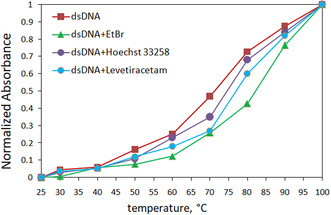
Thermal melting profile of the LEV–dsDNA complex and dsDNA (120 μM).

This experiment plays a crucial role in examining the binding interactions between small molecules and DNA. When subjected to heat, dsDNA undergoes denaturation, transforming into ssDNA as a result of the disruption of base stacking interactions and hydrogen bonds. This process induces various alterations in the DNA structure, including changes in shape, UV absorption, and melting temperature (*T*_m_). The melting temperature is defined as the point at which half of the dsDNA has transitioned into ssDNA following denaturation.^[^ ^35^ ^]^ The interaction of small molecules with DNA can potentially influence the DNA solution's *T*_m_. Specifically, intercalating agents can elevate the *T*_m_ by ≈10 to 12 °C, whereas molecules that bind within the grooves of the DNA exhibit minimal impact on the *T*_m_.^[^ ^36^ ^]^ In this research, the absorbance of the dsDNA solution was measured at 260 nm under different temperature conditions ranging from 20 to 100 °C, with and without LEV. Based on the melting curve midpoints, the *T*_m_ values for dsDNA and the LEV–dsDNA combination were calculated to be 72.2 °C and 77.6 °C, respectively (**Table** **2**). The slight variation in *T*_m_ values provides additional evidence for the groove‐binding interaction between LEV and dsDNA (**Figure** **2**).

**Table 2:** The thermal denaturation points of dsDNA, the LEV–dsDNA complex, and the dsDNA complexes formed with various dyes.

Compound	*T*_m_ (°C)	Δ*T*_m_ (°C)
dsDNA	72.2 °C ± 0.07	–
dsDNA + EtBr	83.4 °C ± 0.08	11.6 °C
dsDNA + Hoechst‐33 258	77.8 °C ± 0.17	5.6 °C
dsDNA + Levetiracetam	77.6 °C ± 0.15	5.4 °C

### 2.4 Fluorescence Study

**Figure 3:**
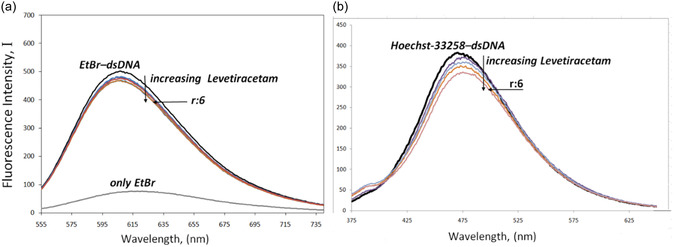
The fluorescence spectra of a) the Hoechst 33 258–dsDNA complex and b) the ethidium bromide–dsDNA complex.

The fluorescence displacement assay serves as a valuable technique for investigating the interaction between DNA and ligands through the application of probe molecules. When ligand molecules successfully displace the probe molecules from their complex with DNA, it indicates that the binding mechanisms of the ligands and the probes are analogous in their interaction with the DNA structure.^[^ ^37^ ^]^ Ethidium bromide (EtBr), a well‐known intercalating agent, exhibits minimal fluorescence in aqueous environments, a phenomenon attributed to the quenching effects of solvent molecules. However, upon intercalating with the base pairs of DNA, the fluorescence level of EtBr significantly increases. Similarly, the groove‐binding agent Hoechst 33 258 also demonstrates an enhancement in fluorescence intensity following its interaction with DNA.^[^ ^38^ ^]^ In order to assess the way that LEV binds to dsDNA, we chose EtBr and Hoechst 33 258 as probe compounds for this investigation. **Figure** **3** shows how LEV affected the fluorescence spectra for the EtBr–dsDNA and Hoechst 33 258–dsDNA complexes. Analysis of this figure reveals a significant reduction in the fluorescence intensity of the Hoechst 33 258‐dsDNA system as the concentration of LEV escalated, whereas the binding of EtBr to dsDNA remained largely unaffected. These findings indicate a competitive interaction between LEV and Hoechst 33 258, thereby reinforcing our earlier observations.

**Figure 4:**
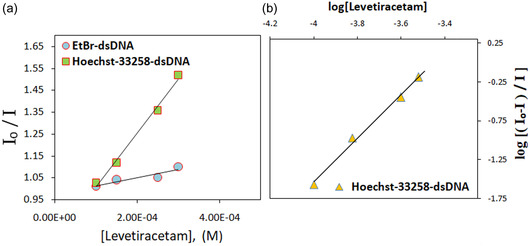
The Stern–Volmer plots illustrate a) the interaction between LEV, dsDNA, and b) the corresponding curve.

To assess the variations in the fluorescence intensity of different dyes conjugated to dsDNA by LEV, the following equation was employed to calculate the Stern–Volmer quenching constant (K_sv_) values from the Stern–Volmer plot (**Figure** **4a**).

(2) $$\frac{I_{o}}{I}{\text{= 1 + }K}_{\text{sv}}\text{[Q]}$$

Here, *I*_o_ stands for the fluorescence level of LEV in the absence of a quencher, while I stands for the intensity when a quencher is present. [Q] stands for the quencher's concentration, and *K*_sv_ is the Stern–Volmer quenching coefficient.

The following formula was used to determine the binding constant (*K*_b_) and the binding stoichiometry (*n*) for each DNA molecule based on the intercept and slope derived from the graph of log (*I_o_‐I/I*) versus log [Q], as illustrated in Figure 4b:^[^ ^39^ ^]^

(3) $$\text{log }\left(\frac{I_{o}\text{ ‐ I}}{I}\right){\text{= log }K}_{b}\text{ + }n\text{ log [Q]}$$

The analysis of the collected data reveals that the Stern–Volmer constant indicates the ability of LEV to effectively quench fluorescence. The *K*_sv_ values recorded for EtBr and Hoechst are 1.17 × 10^2^ M^−1^ ± 0.10 and 1.63 × 10^3^ M^−1^ ± 0.16, respectively. Furthermore, as Figure 4a shows, the K_sv_ value linked to Hoechst was substantially greater than that of EtBr. These findings support the idea that LEV binds inside the dsDNA minor groove. Additionally, it was computed that the stoichiometry of the binding was 1.17 and that the DNA binding constant was 2.57 × 10⁴ M^−1^ ± 0.20.

The inner filter effect is a loss of observed fluorescence intensity caused by the sample's light absorption. The effect is closely linked to the path the light takes through the sample before reaching the detector.^[^ ^40^ ^]^ The Agilent Cary Eclipse fluorescence spectrometer we use in our studies is sensitive, accurate, and flexible. In our studies, the inner filter effect did not interfere with fluorescence measurements. It did not limit the linear dependence of fluorescence signals even at low sample concentrations.

### 2.5 Viscosity Studies

Viscosity serves as a highly precise method for elucidating the binding mode within a solution. Measurements of viscosity exhibit sensitivity to variations in DNA length. Although electrostatic interactions and groove binding modes do not significantly affect the viscosity of DNA solutions, the intercalation mode leads to an elongation of the DNA helix. This elongation occurs due to the separation of base pairs at the intercalation sites, ultimately resulting in an increase in the viscosity of the DNA solution^[^ ^41^ ^]^ Viscosity tests were performed on the dsDNA solutions mixed with the LEV at 25 °C to determine the binding mechanism and the intensity of the interaction between the two molecules. The values of η and η_0_ were calculated using equation (3).

**Figure 5:**
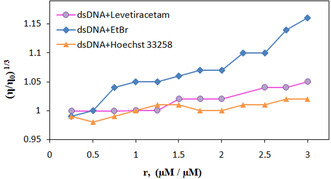
The impact of increasing LEV concentration on the relative viscosity of dsDNA (r = [LEV]/[dsDNA] = 0.25–3.0).

The relative viscosity values, expressed as (η/η_0_)^1/3^, were graphed against the ratio r = [LEV]/[DNA], where η represents the viscosity of dsDNA in the presence of LEV, and η_0_ denotes the viscosity of dsDNA in the absence of LEV. As presented in **Table** **3**, there are no significant alterations in viscosity with the incremental addition of the LEV solution (30−360 μM) (**Figure** **5**). This observation suggests that LEV interacts with dsDNA through a groove‐binding mechanism.^[^ ^42^, ^43^ ^]^ The findings align with the outcomes of the competitive binding analysis regarding the interaction of LEV with dsDNA, specifically through the mechanism of binding within the minor groove.

**Table 3:** Estimated viscosity values and Herschel–Bulkey parameters for LEV.

Sample no.	Yield stress (Pa)	K (Pa.s ^n^)	n (–)	Viscosity (mPa.s)
Buffer	0.32 ± 0.01	5.95 × 10^−5^ ± 4.22 × 10^−5^	1.57 ± 0.02	16.02
LEV	0.32 ± 0.07	1.70 × 10^−4^ ± 3.25 × 10^−5^	1.41 ± 0.06	16.58
DNA	0.32 ± 0.01	8.52 × 10^−5^ ± 2.73 × 10^−5^	1.51 ± 0.02	16.40
Sample 1	0.32 ± 0.05	7.40 × 10^−5^ ± 1.47 × 10^−5^	1.52 ± 0.02	16.35
Sample 2	0.32 ± 0.04	7.00 × 10^−5^ ± 2.24 × 10^−5^	1.54 ± 0.01	16.35
Sample 3	0.32 ± 0.02	7.85 × 10^−5^ ± 3.14 × 10^−5^	1.53 ± 0.02	16.38
Sample 4	0.32 ± 0.07	1.10 × 10^−4^ ± 1.78 × 10^−5^	1.46 ± 0.04	16.44
Sample 5	0.32 ± 0.04	1.35 × 10^−4^ ± 6.81 × 10^−5^	1.45 ± 0.05	16.52
Sample 6	0.32 ± 0.05	1.75 × 10^−4^ ± 2.71 × 10^−5^	1.41 ± 0.03	16.60
Sample 7	0.32 ± 0.07	1.50 × 10^−4^ ± 5.21 × 10^−5^	1.46 ± 0.01	16.60
Sample 8	0.32 ± 0.06	1.40 × 10^−4^ ± 1.87 × 10^−5^	1.49 ± 0.03	16.61
Sample 9	0.32 ± 0.03	2.11 × 10^−4^ ± 1.52 × 10^−5^	1.38 ± 0.04	16.67
Sample 10	0.33 ± 0.02	3.95 × 10 ^−5^ ± 1.56 × 10 ^−5^	1.62 ± 0.02	16.69

### 2.6 Electrochemical Studies

**Figure 6:**
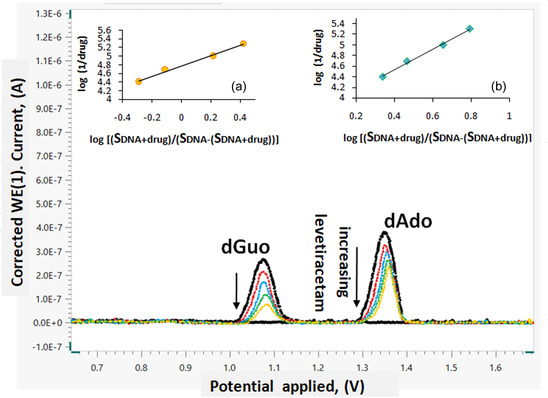
Differential pulse voltammograms of the mixture of LEV–dsDNA (in pH 4.8 acetate buffer; fixed 50 μM dsDNA concentration, and different LEV solutions 5−40 μM).

Complexes are created as a result of intricate relationships between DNA and tiny molecules, which alter the quantities of target molecules and free DNA in solution. These alterations subsequently lead to fluctuations in the oxidation states of dsDNA. Therefore, electrochemical techniques are frequently used to monitor and examine how medicines and DNA interact.^[^ ^44^ ^]^ This study investigated the electrochemical interactions between LEV and dsDNA utilizing a glassy carbon electrode (GCE). As increasing LEV concentrations were added to the dsDNA solution, differential pulse voltammogram (DPV) was utilized to determine the oxidation signals. As shown in **Figure** **6**, the oxidation of deoxyguanosine (dGuo) and deoxyadenosine (dAdo) at potentials of +1.09 V and +1.36 V, respectively, were responsible for the two different peaks seen in the dsDNA solution's DPV voltammogram. Importantly, the addition of LEV decreased the current values of the dGuo and dAdo peaks, suggesting a strong correlation between LEV and dsDNA.

The binding constants (*K*_b_) of LEV were determined using the equation outlined below, which the observed decrease in peak heights resulting from the interaction with dsDNA:^[^ ^45^ ^]^

(4) $$\text{log}\left(\frac{1}{\left[\text{Drug}\right]}\right){\text{= log}K}_{b}\text{ + log}\left(\frac{S_{\text{dsDNA‐drug}}}{S_{\text{dsDNA }}{\text{‐ S}}_{\text{dsDNA‐drug}}}\right)$$

Here, [drug] stands for the concentration of LEV, S_dsDNA_ signifies the current that is solely attributed to dsDNA, and S_dsDNA‐drug_ for the current that results from the association of LEV and dsDNA.

Analysis of the results showed that LEV interacted with guanine and adenine. It was found that the binding caused a very minor change in the bases’ oxidation potential values in the direction of positive potential. The slope and intercept of the curve were used to calculate the electrochemical DNA binding constant of LEV. Guanine and adenine bases have binding constant values of 6.02 × 10^4^ M^−1^ ± 0.21 and 5.75 × 10^3^ M^−1^ ± 0.32, respectively, based on calculations. The values of the binding constants obtained using different approaches are quite similar to those obtained using electrochemical methods. While the compounds participating in groove binding have binding constants ranging from 10^3^ to 10^4^, the intercalated compounds exhibit values close to 10^7^. These findings imply that the interaction between LEV and dsDNA occurs in a specific manner, predominantly through groove binding.

### 2.7 Computational Studies

#### 2.7.1 Geometry Optimization and MEP (Molecular Electrostatic Potential) Map Calculations

This study used density functional theory (DFT) calculations to optimize the geometry of the LEV molecule in water and compute its MEP map prior to molecular docking analysis. B3LYP functional with a 6‐311 + G(d,p) basis set was chosen for the calculations as it is known to provide a good balance between accuracy and computational efficiency for organic molecules. Geometry optimization was performed to ensure that the molecule was in its lowest energy conformation, minimizing the influence of any steric clashes or unfavorable bond angles. This step is critical for obtaining reliable results in subsequent docking studies, as the conformation of the molecule affects its interaction with dsDNA. In addition, the MEP map of LEV was calculated to visualize the charge distribution on the surface of the molecule. This map helps to understand the negative and positive regions of the molecule that are most likely to interact with dsDNA, providing insight into potential binding sites. The electrostatic potential analysis also highlights polarity and charge distribution, which are essential factors in evaluating LEV–dsDNA interactions. The results of DFT calculations serve as a foundational step to ensure that the molecule is stable and well‐defined before performing the molecular docking calculations to predict its interactions with dsDNA.

**Figure 7:**
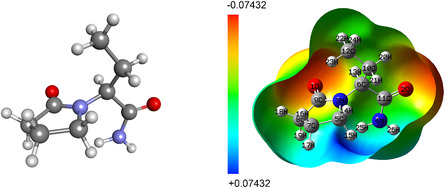
Optimized geometry and MEP map of LEV.

The optimized geometry and MEP map of the LEV molecule are given in **Figure** **7**. The results show that the negative charge is concentrated on the oxygen atoms as expected, while the positive charge is concentrated mainly on the –NH_2_ group hydrogens. In addition, as explained in the next step, it was observed that these positive centers act as hydrogen bond donors in the formation of hydrogen bonds between LEV and dsDNA.

#### 2.7.2 Molecular Docking Studies

**Figure 8:**
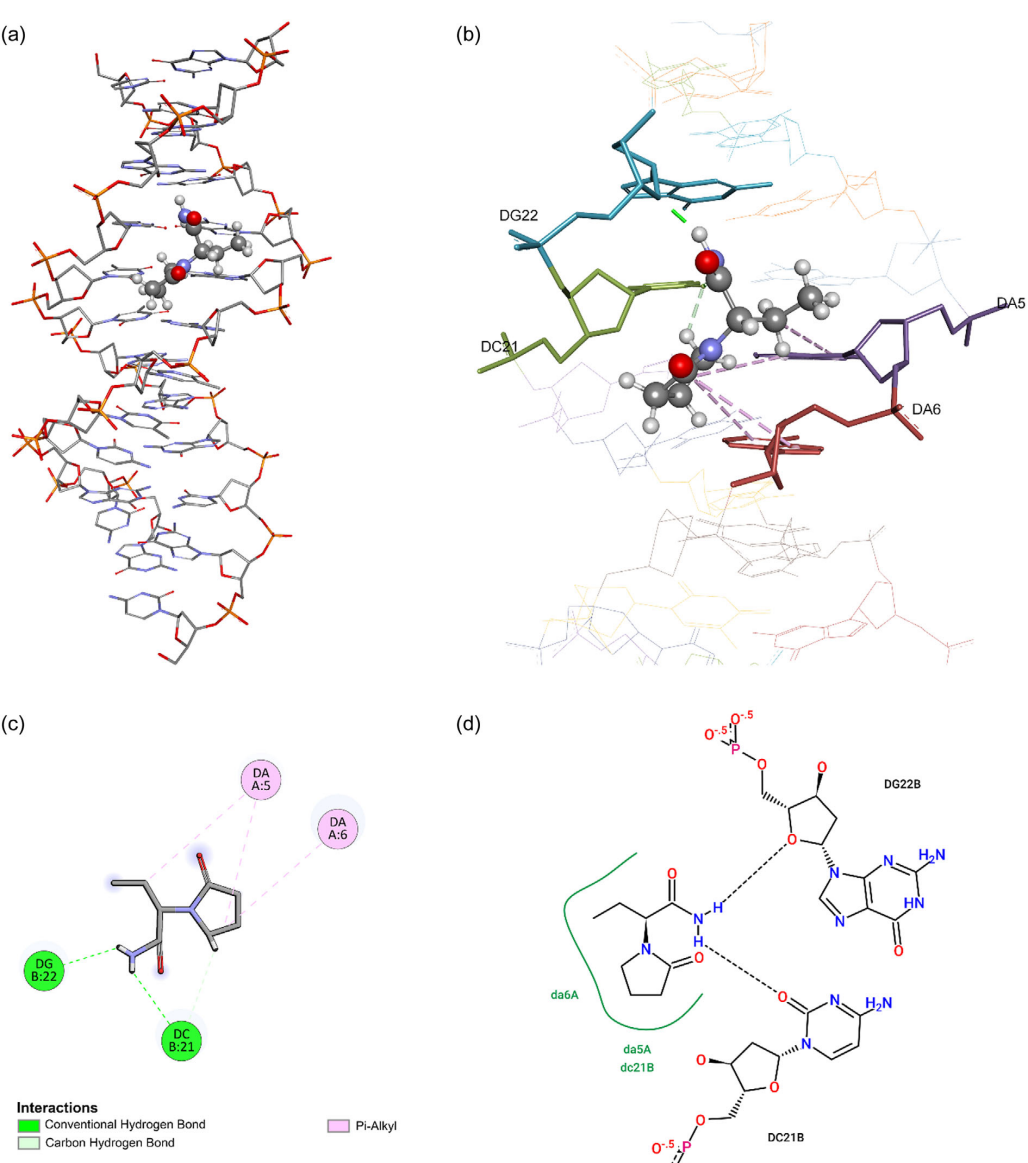
a) LEV–dsDNA binding pose, b) LEV–dsDNA interactions, c) LEV–dsDNA interactions (2D representation), and d) LEV–dsDNA hydrogen bonds (2D representation).

Molecular docking studies were carried out to investigate the interactions between LEV and dsDNA, specifically identifying the binding site and the nature of interactions. Molecular docking results revealed that LEV binds to the minor groove of dsDNA, which is a common binding site for many small molecules and drugs targeting nucleic acids. The significant interactions between LEV and dsDNA were found to be hydrogen bonds and π‐alkyl interactions. These noncovalent interactions play a crucial role in stabilizing the LEV–dsDNA complex. Hydrogen bonds were observed between –NH_2_ group hydrogens of LEV and the guanine (DG22) and cytosine (DC21) bases of dsDNA, while adenine (DA5 and DA6) bases took a role through π‐alkyl interactions (**Figure** **8**). The preferential binding to the minor groove suggests that LEV may act as a groove binder, which is a characteristic often associated with compounds that can modulate DNA structure or interfere with its function.

### 2.8 Quantification Studies

**Figure 9:**
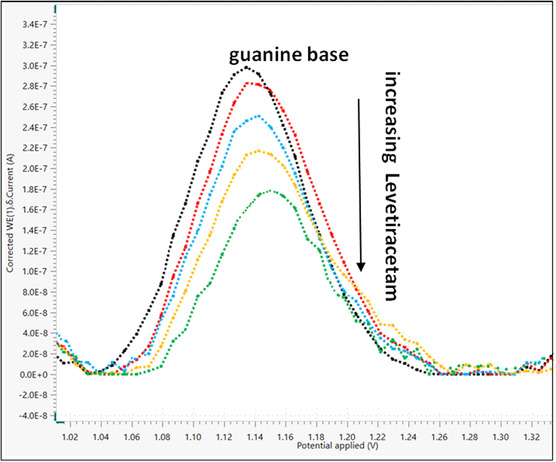
Modifications to the dsDNA current signal resulted from the inclusion of LEV employing the DPV method.

This research introduced a novel methodology for quantitative analysis through the interaction of LEV with dsDNA. This marks the inaugural development and validation of such an approach for LEV quantification. The calibration curve was established by assessing the variations in the oxidation current peak of the dGuo in response to varying concentrations of LEV (**Figure** **9**). Oxidation signals were captured in a pH 4.8 acetate buffer medium using the DPV technique, with specific conditions set for the potential scan range (from 0.0 V to +1.8 V), scan rate (0.01 V/s), step potential (8 mV), modulation amplitude (50 mV), modulation time (0.05 s), and interval time (0.5 s). The validation parameters were computed once the calibration curve was established.

Considering the aforementioned details, an electroanalytical method for quantifying LEV can be developed based on the data obtained from dGuo concerning the interaction between LEV and dsDNA. The optimal supporting electrolyte for this voltammetric study was identified as a 0.50 M acetate buffer at pH 4.80, supplemented with 0.02 M NaCl, as this combination yielded the best peak response while minimizing background noise. The relationship between current and concentration was established by observing the reduction in peak currents associated with the oxidation of dGuo in response to varying LEV concentrations and its binding affinity to dsDNA, utilizing a GCE. A linear calibration curve was generated when the dGuo current values were plotted against LEV concentrations ranging from 2.5 μM to 20 μM. The calibration equation can be represented as I (A) = −5 × 10^−9^ C (μM) + 3 × 10^−7^, with a correlation coefficient of R^2^ = 0.9700. Limit of detection (LOD) values (LOD = 3 × SD/slope) and limit of determination (LOQ) values (LOQ = 10 ×  SD/slope) for the specified concentration range were determined through regression analysis, yielding values of 0.70 μM and 2.31 μM, respectively.^[^ ^46^ ^]^

The accuracy of the method was investigated in multiple reference solutions throughout the course of one single day in order to assess the intraday repeatability of voltammetric assessment. Every time, a specific concentration of newly generated solutions was used. On the other hand, the interday repeatability was evaluated by repeating the measurement over the course of a month. Following the interaction with LEV‐dsDNA, the intraday and interday repeatability calculations were performed, yielding relative standard deviation (RSD%) values of 1.87 and 2.26, respectively, as presented in **Table** **4**.

**Table 4:** Calibration curve validation parameters for the LEV–dsDNA interaction derived from the dGuo signal using the DPV technique.

Measured potential	1.13 V
Linearity range	2.5–20 μM
Slope (A/μM)	−5 × 10^−9^
Intercept (A)	3 × 10^−7^
Correlation coefficient	0.9700
Limit of detection (LOD)	0.70 μM
Limit of determination (LOQ)	2.31 μM
Intra‐day repeatability (%RSD)	1.87
Inter‐day repeatability (%RSD)	2.26

### 2.9 Analytical Applications of the Developed Voltammetric Method

A quantitative assessment of the commercial preparation of LEV was performed through the established % recovery studies, employing a method that we have developed and rigorously validated. One of the primary advantages of this analytical technique is that it eliminates the need for time‐consuming pretreatment of samples, which often requires various steps prior to analysis. Following these findings, the proposed method for LEV quantification, which utilizes the dsDNA binding technique, was applied to determine the concentration of LEV in Keppra 250 mg tablets (**Table** **5**). Quantification studies were carried out under the same conditions as those used for standard solutions, involving the acquisition of DPV voltammograms with specific parameters: a potential scan range from 0.0 V to +1.8 V, a scan rate of 0.01 V/s, a step potential of 8 mV, a modulation amplitude of 50 mV, a modulation time of 0.05 s, and an interval time of 0.5 s. Solutions were prepared from the LEV tablet to align with the linearity of the corresponding calibration curve. The analytical accuracy of the method in a pH 4.8 medium was evaluated by calculating the bias percentage between the calculated mean concentration and the true concentration, while the calculated RSD of less than 5% further substantiates the reliability of the technique.

**Table 5:** Findings from the study conducted with the Keppra LEV tablet in an acetate buffer with a pH of 4.8.

Sample no.	Amount of substance in tablet (250 mg)
1	246
2	247
3	247
4	251
5	253
Average	249
SD	3.03
% RSD	1.22

We have successfully performed percentage recovery experiments to assess whether the excipients present in pharmaceutical formulations of the drug affect the quantification process. A particular concentration of calibration curve was selected, and solutions were prepared for these investigations that contained a certain amount of pure LEV substance and medicine from the tablet dosage form. The voltammograms of these solutions were captured under the same conditions as those used for thestandard preparations. The findings presented in **Table** **6** confirm that the components do not interfere with the interaction between LEV and dsDNA. Consequently, it can be concluded that the proposed method is dependable, precise, and adequately specific for application with commercial formulations.

**Table 6:** Results of the percentage recovery from a 250 mg Keppra tablet containing LEV in a pH 4.8 acetate buffer.

Amount of substance added (mg)	Amount of substance found (mg)	% recovery
0.10	0.09	90
0.10	0.08	80
0.10	0.08	80
0.10	0.11	110
0.10	0.12	120
		Average: 96
		SD:18.16
		%RSD: 18.92
		%Bias:−4

### 2.10 Comparison with Other Methods

A comprehensive review of the scientific literature revealed multiple established techniques for the determination of LEV. Chromatographic techniques frequently offer high accuracy, sensitivity, and isolation. Nevertheless, they need extensive organic solvent and chemical procedures like preanalysis modification. Furthermore, these methods tend to be more costly compared to alternative techniques, necessitate longer analysis times, and are often unsuitable for routine or localized research applications. In contrast, electroanalytical methods, particularly voltammetric techniques, present a viable alternative due to their numerous advantages, including user‐friendliness, affordability, rapid execution, sufficient sensitivity, and reduced reliance on hazardous chemicals. In this context, we have developed a novel method for quantifying LEV based on its interaction with dsDNA utilizing DPV. This newly devised technique offers several benefits over traditional methods, such as simplicity, quick analysis times, cost‐effectiveness, and environmental sustainability, enabling rapid, straightforward, and precise detection of pharmaceutical samples. A comprehensive overview of the various techniques for assessing LEV is presented in **Table** **7**.

**Table 7:** Comparing analytical techniques developed for determining LEV.

Methodology	Linear range	LOD	Sample	Recovery (%)	Ref.
UV	2–10 μg/mL	0.0141 μg/mL	Tablet	99.53	[59]
HPLC–UV	1–60 μg/mL	0.25 μg/mL	Plasma	97.15	[60]
HPLC–UV	4–80 μg/mL	1.0 μg/mL	Plasma	90.0	[28]
GC/MS	0.50–50.0 μg/mL	0.15 μg/mL	Blood	90.0	[26]
Electrochemical	0.10–0.83 mM	10.39 μM	Plasma and tablet	98.14	[61]
DPV	2.5–20 μM	0.70 μM	Tablet	96	This study

## 3 Conclusion

The initial part of the study focused on elucidating the interaction mechanism between LEV and dsDNA. Various spectroscopic, thermodynamic, and computational techniques were employed to investigate how LEV binds to dsDNA. The phenomenon of hyperchromism observed in the UV–vis absorption was attributed to conformational alterations within the dsDNA structure. The binding constant (*K*_b_) values derived from fluorescence spectroscopy indicated a significant affinity of LEV for dsDNA, with the binding constant measured in the range of 10^4^ M^−1^, suggesting a groove–binding interaction. Results from displacement and viscosity experiments supported the conclusion that LEV associates with the dsDNA groove, a finding further validated by docking studies. The insights gained from the interaction between LEV and dsDNA are expected to contribute to a better understanding of the pharmacological properties of this therapeutic agent.

A novel methodology was developed to explore the interaction between LEV and dsDNA. We conducted quantification tests and percent recovery assessments using pharmaceutical tablets based on our innovative technique. The impressive outcomes achieved through the DPV technique demonstrate that our methods are efficient, rapid, and noninvasive. Furthermore, the process does not require intricate equipment or extensive sample preparation. The high recovery rates suggest that the additives present in the formulations do not interfere with the determination of LEV using our methods. Consequently, this approach can be effectively applied to biological samples such as blood, urine, and milk for routine quality assessments of LEV in commercial preparations.

## 4 Experimental Section

4.1

4.1.1

##### Reagents

The LEV chemical was obtained from Deva Holding in Istanbul, Turkey, while the Keppra 250 mg tablet was purchased from a local pharmacy. All solvents used in this research were procured from Merck, and additional reagents such as tris(hydroxymethyl)aminomethane hydrochloride (≥99.9%), sodium chloride (≥99.0%), double‐stranded DNA from fish sperm (≥99.0%, CAS Number: 100 403‐24‐5), acetic acid (99.0%), sodium acetate (≥99.0%), ethidium bromide (EtBr), and Hoechst 33 258 (≥98%) were sourced from Sigma‐Aldrich. Each of these reagents and solvents was of reagent grade and utilized directly from their original containers, eliminating the need for any further purification or processing.

##### Instrumentations

The absorption spectra were obtained using a T80 + UV/VIS spectrophotometer (PG Instruments Limited, UK) fitted with a 1 cm quartz cell. A Cary Eclipse fluorescence spectrometer (Agilent Technologies, USA) was used for each fluorescence study. An Autolab potentiostat/galvanostat (PG‐STAT 302 N, Eco Chemie, Netherlands) and an electrochemical apparatus intended to run a three‐electrode cell were used for the voltammetric tests. An Ag/AgCl electrode was used as the reference electrode, a platinum wire was used as the auxiliary electrode, and a GCE with a diameter of 3.0 mm was used as the working electrode in the electrochemical experiments. NOVA 2.1.7 software was used on a computer to control the device. A rheometer (HAAKE RheoStress 1, Germany) equipped with a parallel plate sensor (35 mm diameter and 1 mm gap) was used to measure the viscosity of different samples. The experimental setup also required a programmable heater (Heidolph MR 3001 K) and a Seven Excellence DO meter S600 (Mettler Toledo, UK).

##### Sample Preparation

Distilled water served as the solvent for the preparation of stock solutions of LEV at a concentration of 1 mM and dyes at 5 mM. The dsDNA was prepared by dissolving several DNA strands in a solution that included 150 mM NaCl and 0.2 M Tris‐HCl at pH 7.4. In order to attain uniformity, this mixture was then kept at 4 °C for a period of at least 24 h, during which it was gently agitated periodically. The mixture was quickly stirred using a lab shaker to produce a homogeneous solution. The concentration of dsDNA per nucleotide was determined using a mean extinction coefficient value of 6600 M^−1^ cm^−1^ at A_260_ nm.^[^ ^46^ ^]^ The dsDNA was found to have a reasonable degree of purity, as shown by the absorbance ratio of 1.87 at 260 and 280 nm. To create working solutions, a dilution procedure according to the experimental requirements was used, with all procedures conducted in a 10 mM Tris‐HCl buffer at pH 7.4.

##### Methods: UV–vis Spectroscopy

The absorption spectra of DNA at a concentration of 150 μM, both in the presence and absence of LEV at concentrations ranging from 25 to 300 μM, were obtained using a UV–visible spectrophotometer equipped with a quartz cuvette (1 cm). The analysis was conducted at room temperature over a 200–400 nm wavelength range.

##### Methods: Thermodynamic Parameters

This work aimed to elucidate the thermodynamic characteristics of the binding process between dsDNA and LEV. The Vant Hoff equation was used to determine these thermodynamic parameters.

(5) $$\text{In}K_{b}=-\frac{\Delta H}{\text{RT}}+\frac{\Delta S}{R}$$

In this case, T stands for the temperature in kelvin, R for the gas constant, and *K*_b_ for the binding constant.^[^ ^47^ ^]^ The following formula can be used to determine the change in Gibbs’ free energy (Δ*G*)

(6) ΔG=ΔH−TΔS

Three different temperatures (288, 298, and 308 K) were used for the experiment to guarantee the most accurate results and find any signs of non‐linearity.

##### Methods: DNA Melting Temperature (*T*_m_) Study

The melting temperatures of dsDNA and the LEV–dsDNA complex were compared using a spectrophotometer paired with a thermocouple. At temperatures ranging from 20 to 100 °C, the absorbance at 260 nm was measured for dsDNA (120 μM) and the LEV–dsDNA complex (1:12 ratio). Following that, a plot of the absorbance measurements of samples against temperature changes was created. The melting curves’ halfway point served as the basis of the *T*_m_ values.

##### Methods: Fluorescence Studies

Our work used two well‐known fluorescent probes, ethidium bromide (EtBr), which functions as an intercalator, and Hoechst 33 258, a groove binder, to ascertain the binding process of LEV to dsDNA. We titrated the dye–dsDNA complexes at a 1:10 ratio in various experimental setups with LEV concentrations ranging from 50 to 300 μM. The excitation wavelengths for the dye–dsDNA complexes were set at 343 nm for Hoechst 33 258 and 530 nm for EtBr, and the resulting fluorescence emission spectra were meticulously recorded for analysis.

##### Viscosity Measurements

Viscosity experiments were conducted utilizing a viscometer maintained within a constant temperature bath set at 25 °C. The flow times of the Tris‐HCl buffer and a 120 μM dsDNA solution were recorded following the incremental addition of LEV, with measurements taken using a digital stopwatch. Each testing solution underwent three separate measurements to ensure accurate viscosity values, calculated using the formula

(7) $$\eta =\frac{{\text{t ‐ t}}_{0}}{t_{0}}$$

Where t_0_ represents the flow time of the Tris‐HCl buffer and t denotes the flow time of the dsDNA solution with varying concentrations of LEV, ranging from 30 to 360 μM. Ultimately, the collected data were represented graphically as (*η/η*_0_)^1/3^ plotted against r, with *η*_0_ and η indicating the viscosity of dsDNA in the absence and presence of LEV, respectively.

##### Methods: Voltammetric Measurement

In acetate (pH 4.8) buffer, DPV investigations were performed in the potential range from +0.8 V to +1.6 V. For the quantification of LEV and its interaction with DNA, the measurements were executed within specific ranges for step potential (0.005 V to 0.1 V), modulation amplitude (0.01 V to 0.12 V), modulation time (0.01 s to 0.12 s), and interval time (0.1 s to 1.0 s). These parameters represent the optimal settings for the DPV measurements, where one variable is altered while the others remain fixed. Before each measurement, the GCE underwent a thorough cleaning process using an alumina slurry (particle size of 0.05 μm) applied on a polishing pad. A constant quantity of dsDNA at 50 μM was mixed with different quantities of LEV (from 5 to 40 μM) in order to calculate the binding constant. The voltammograms obtained were processed using a moving average technique to perform baseline correction after each measurement.

##### Analysis of a Marketed Formulation

The experiment involved carefully weighing, pulverizing, and appropriately mixing five Keppra pills, each having 250 mg of LEV. The resulting powder, corresponding to a concentration of 1 mM LEV, was then transferred into a 100 mL volumetric flask, which was combined with a pH 4.8 acetate buffer solution. This mixture underwent ultrasonic treatment for 30 min to ensure complete dissolution. Following this, the solution was filtered to eliminate any particulates and subsequently utilized to create additional experimental solutions. To assess the effectiveness of the procedure, recovery tests were conducted by introducing a known quantity of pure LEV into the tablet solution, allowing for the evaluation of the active ingredient's concentration through a calibration curve derived from regression analysis.

##### Computational Studies: Geometry Optimization and MEP Map Calculations

In the preliminary part of the computational studies, geometry optimization and MEP map calculations were performed on the LEV molecule. The initial geometry of LEV was obtained from the PubChem database. Then, geometry optimization was carried out using the B3LYP (Becke three‐parameter hybrid functional combined with Lee‐Yang‐Parr correlation functional), the 6‐311 + G(d,p) basis set, and the IEFPCM (the polarizable continuum model using the integral equation formalism variant) solvation model with water as the solvent. A frequency analysis was performed after geometry optimization to validate that the geometry‐optimized structure corresponds to a real minimum. The map of the optimized geometry's MEP was then determined. DFT calculations performed in this part of the study were carried out with the use of Gaussian 09 Rev. D01^[^ ^49^ ^]^ and GaussView5^[^ ^50^ ^]^ Software packages.

##### Computational Studies: Molecular Docking Studies

The dsDNA fragment required for the molecular docking calculations was obtained from the RCSB Protein Data Bank^[^ ^51^ ^]^ (PDB ID: 1BNA) (Resolution: 1.90 Å, R‐Value (DCC): 0.220, R‐Value (Depositor): 0.178) while the structure of LEV was obtained from the DFT calculations performed in the previous step. Before molecular docking calculations, water molecules were removed from the dsDNA structure, hydrogens and Gasteiger charges were added, and nonpolar hydrogens were incorporated. Molecular docking calculations were performed in an 18 × 20 × 26 Å^3^ grid box. AutoDock Tools^[^ ^52^ ^]^ and AutoDock Vina^[^ ^53^ ^]^ software packages were used in molecular docking studies, while Discovery Studio Visualizer^[^ ^54^ ^]^ and PoseView^[^ ^55^, ^56^, ^57^, ^58^ ^]^ were used to visualize the results.

## Conflict of Interest

The authors declare no conflict of interest.

## Data Availability

The data that support the findings of this study are available from the corresponding author upon reasonable request.
